# Neuropsychiatric Manifestations of a Frontal Lobe Meningioma: A Case Report

**DOI:** 10.7759/cureus.68101

**Published:** 2024-08-29

**Authors:** Syed Ali Bokhari, Muhanad Elnoor, Alma Al Mansour, Khalid Mustafa, Abdelaziz Osman

**Affiliations:** 1 Psychiatry, Al Amal Psychiatric Hospital, Emirates Health Services, Dubai, ARE; 2 Emergency Medicine, Al Qassimi Hospital, Emirates Health Services, Sharjah, ARE; 3 General Practice, Al Amal Psychiatric Hospital, Emirates Health Services, Dubai, ARE

**Keywords:** depression, neuroimaging, frontal meningioma, brain tumors cns tumors, neurology, psychiatry, neuropsychiatry, psychosis, meningioma

## Abstract

The exclusion of organic causes for psychiatric symptoms is a routine practice in mental healthcare. Brain tumors can elicit a range of mood, behavioral, or cognitive symptoms that mimic mental health disorders, significantly altering a patient's personality and behavior if left undiagnosed or untreated. This case report presents a 56-year-old Middle Eastern male with no prior history of mental illness who exhibited a three-week history of depressive symptoms, social withdrawal, and poor self-care. Despite treatment, his condition deteriorated, manifesting psychomotor retardation, urinary incontinence, paranoia, mood lability, and sexually disinhibited behavior. Neuroimaging revealed a large extra-axial mass in the anterior cranial fossa, indicative of a meningioma, necessitating referral to neurosurgery. CT and MRI scans confirmed a hyperdense mass lesion (7.1 x 7.7 x 7.5 cm), causing structural erosion and a midline shift. This case underscores the importance of considering organic causes in atypical psychiatric presentations. Meningiomas, particularly those in the frontal lobes, can present primarily with psychiatric symptoms, complicating early diagnosis. Neuroimaging is critical for accurate diagnosis and effective management in such cases. Clinicians should be vigilant for organic causes in patients with atypical psychiatric symptoms, especially in those over 50. Early neuroimaging can lead to timely diagnosis and treatment, significantly improving patient outcomes.

## Introduction

In mental healthcare, it is routine and essential to rule out organic causes when assessing psychiatric symptoms. Organic causes, referring to physical conditions affecting the brain, can include space-occupying lesions such as brain tumors. These tumors are capable of inducing a wide spectrum of psychiatric symptoms, ranging from mood disturbances to significant behavioral changes and cognitive impairment. These symptoms, known as neuropsychiatric or neurobehavioral symptoms, can sometimes closely mimic or overlap with primary psychiatric disorders. If these symptoms are not promptly identified and treated, they can lead to profound alterations in the patient's personality, mood, and overall behavior [1].

The prevalence of psychiatric symptoms among patients diagnosed with brain tumors is notable, with studies indicating that between 50% and 78% of these patients initially present with such symptoms [2]. Meningioma, the most common non-malignant brain tumor, also ranks as the second most common tumor of the central nervous system overall [3]. In some cases, brain tumors may present without obvious neurological signs, with psychiatric symptoms being the sole indication of an underlying condition. Given this, neuroimaging techniques are crucial for clinicians when organic causes, such as brain tumors, need to be ruled out, particularly in patients presenting with atypical, newly emerged, or challenging-to-treat symptoms [4,5].

We report the case of a late middle-aged man who was brought to psychiatric services for the first time with depressive symptoms. In a short span of time, his condition deteriorated to include increasing suspiciousness, persecutory delusions, mood instability, significant mood swings, sexually disinhibited behavior, and irritability. Given concerns about potential organic causes, considering the patient's age, the sudden onset of a psychotic episode without any prior psychiatric history, and the absence of a family history, neuroimaging was performed. This revealed a large frontal lobe meningioma.

This case is noteworthy, as such presentations of frontal lobe meningiomas initially presenting with psychiatric symptoms are infrequently reported in the literature within Middle Eastern, Gulf, or Arab countries, emphasizing the importance of recognizing and documenting these occurrences.

## Case presentation

A 56-year-old Middle Eastern male with no prior history of mental illness was brought to the emergency department of a tertiary psychiatric hospital in the United Arab Emirates by his friends, presenting with a three-week history of depressed mood, social withdrawal, low energy, and neglect of personal hygiene. His colleagues had noticed his mobile phone had been off for three weeks and found his apartment in a state of disarray, with food and drink remnants attracting cockroaches. Upon arrival, the patient appeared disheveled and unkempt, reflecting a marked decline in self-care.

His initial vitals were as follows: his temperature was 36.4 °C, his heart rate was 94 beats per minute, his respiratory rate was 18 breaths per minute, his blood pressure was 166/98 mmHg, and his oxygen saturation (SpO_2_) was 100%.

Upon history taking, the patient reported significant difficulties at work that had led him to cease attending his job, accompanied by pervasive feelings of helplessness, hopelessness, and worthlessness. Additionally, he described an inability to muster energy to get out of bed, slowed thinking, increased indecisiveness, slowed movements, and decreased appetite over the same period.

During the mental status examination (MSE) on admission, the patient appeared disheveled with severe psychomotor retardation. Although coherent and relevant, his speech was slow and low in volume. He expressed a depressed mood and exhibited a blunted affect but maintained a logical thought process, denying any delusions, hallucinations, or any other perceptual disturbances. The physical examination was insignificant, with no focal neurological deficits noted. Routine blood and urine investigations were conducted, including a negative COVID-19 test (Table 1).

**Table 1:** Laboratory investigation results on admission. x10³/mcL: thousand cells per microliter; x10⁶/µL: million cells per microliter; g/dL: grams per deciliter; %: percentage; fL: femtoliters; pg: picograms; g/dL: grams per deciliter; x10³/mcL: thousand cells per microliter; mmol/L: millimoles per liter; µmol/L: micromoles per liter; IU/L: international units per liter; U/L: units per liter; pmol/L: picomoles per liter; µIU/mL: micro-international units per milliliter; mIU/L: milli-international units per liter; mm/hr: millimeters per hour; mg/L: milligrams per liter; N/A: not applicable

Group	Detail	Value With Units	Flags	Normal Range
Complete Blood Count	White Blood Cell	8.51 x 10³/mcL	-	4.00-10.00
	Red Blood Cell	4.68 x 10⁶/µL	-	4.50-5.50
	Hemoglobin	15.40 g/dL	-	13.00-17.00
	Hematocrit	44.10%	-	40.00-50.00
	Mean Corpuscular Volume	94.20 fL	-	80.00-100.00
	Mean Corpuscular Hemoglobin	32.80 pg	High	27.00-32.00
	Mean Corpuscular Hemoglobin Concentration	34.80 g/dL	High	31.50-34.50
	Red Cell Distribution Width	14.60%	High	11.50-14.50
	Platelet Count	194.00 x 10³/mcL	-	150.00-450.00
	Mean Platelet Volume	9.0 fL	-	8.0-11.0
	Neutrophils Percentage	62.9%	-	40.0-80.0
	Lymphocytes Percentage	29.90%	-	20.00-40.00
	Monocytes Percentage	5.0%	-	2.0-10.0
	Eosinophils Percentage	1.4%	-	1.0-6.0
	Basophils Percentage	0.80%	-	0.00-2.00
	Neutrophils Absolute Count	5.4 x 10³/mcL	-	2.0-7.0
	Lymphocytes Absolute Count	2.54 x 10³/mcL	-	1.00-3.00
	Monocytes Absolute Count	0.43 x 10³/mcL	-	0.20-1.00
	Eosinophils Absolute Count	0.12 x 10³/mcL	-	0.02-0.50
	Basophils Absolute Count	0.07 x 10³/mcL	-	0.00-0.10
Dialysis Electrolyte & Renal Profile	Sodium Level	142 mmol/L	-	136-145
	Potassium Level	4.00 mmol/L	-	3.50-5.10
	Chloride Level	105 mmol/L	-	98-107
	Carbon Dioxide Level	27.1 mmol/L	-	21.0-32.0
	Anion Gap	10 mmol/L	Low	12-20
	Creatinine	88 µmol/L	-	62-115
	Uric Acid	380 µmol/L	-	208-428
	Random Glucose Level	9.0 mmol/L	High	4.1-5.9
	Urea Level	4.10 mmol/L	-	2.50-6.40
	Estimated Glomerular Filtration Rate	84 mL/min/1.73 m²	-	-
Liver Profile	Total Protein	68 g/L	-	64-82
	Albumin Level	38.4 g/L	-	34.0-50.0
	Total Bilirubin	7.1 µmol/L	-	3.0-17.0
	Alanine Aminotransferase	46 IU/L	-	30-65
	Aspartate Aminotransferase	21 U/L	-	15-37
	Alkaline Phosphatase	61.00 IU/L	-	46.00-116.00
	Gamma-Glutamyl Transferase	49 IU/L	-	15-85
	Amylase Level	54 IU/L	-	25-115
Blood Gases	Fraction of Inspired Oxygen	100%	-	-
Anemia Profile	Vitamin B12 Level	272.0 pmol/L	-	141.0-489.0
Lipid Profile	Total Cholesterol	6.29 mmol/L	N/A	-
	Triglycerides	6.19 mmol/L	N/A	-
	High-Density Lipoprotein (HDL)	0.85 mmol/L	-	0.75-1.85
	Low-Density Lipoprotein (LDL)	3.45 mmol/L	N/A	-
Miscellaneous Chemistry	25-Hydroxy Vitamin D	28.89 nmol/L	N/A	-
Hormones	Prolactin	153.18 µIU/mL	-	45.00-375.00
Thyroid Function	Free Thyroxine (T4)	13.94 pmol/L	-	11.50-22.70
	Thyroid-Stimulating Hormone	1.59 mIU/L	-	0.55-4.78
Serum Toxicology	Ethanol Level	<1.00 mmol/L	-	0.00-86.80
HIV Screening	HIV Antibodies (Type 1 & 2) and p24 Antigen	0.0950 Index	N/A	-
	HIV Antibodies (Type 1 & 2) and p24 Antigen Interpretation	Non-Reactive	N/A	-
Hepatitis Profile	Hepatitis B Surface Antigen	0.15 Index	N/A	-
	Hepatitis B Core Antibody	Non-Reactive	-	-
	Hepatitis B Core Immunoglobulin M	Non-Reactive	N/A	-
	Hepatitis B e Antigen	0.0100	N/A	-
	Hepatitis B e Antibody	Non-Reactive	N/A	-
	Hepatitis C Antibody	0.11 Index	N/A	-
	Hepatitis B Surface Antibody	<3.100 mIU/mL	N/A	-
	Hepatitis B Surface Antibody Interpretation	Non-Reactive	N/A	-
	Hepatitis C Antibody Interpretation	Non-Reactive	N/A	-
Syphilis	Rapid Plasma Reagin	Non-Reactive	-	-
Other Hematology	Erythrocyte Sedimentation Rate	8.00 mm/hr	-	0.00-20.00
Cardiac Profile	Total Creatine Kinase	124 IU/L	-	39-308
Infectious Disease	C-Reactive Protein	1.4 mg/L	-	0.0-3.0
Virology	COVID-19 Polymerase Chain Reaction (PCR)	Not Detected	-	-

The patient was admitted to the general adult psychiatric inpatient ward for further evaluation and treatment. During his hospital stay, he was assessed by a multidisciplinary inpatient team. He was initially diagnosed with major depressive disorder and commenced on escitalopram 10 mg daily and lorazepam 1 mg thrice daily. Despite treatment, he exhibited persistent psychomotor retardation, monotonous and sparse speech, flat affect, poor self-care, and urinary incontinence. Although he was depressed, he denied self-harm or suicidal thoughts. Over the course of his stay, he was moving using a wheelchair as he continued to exhibit psychomotor retardation. He needed assistance for all his activities of daily living (ADLs).

After a few days, the patient's condition evolved. He began to exhibit increasing suspiciousness, initially believing the nursing staff were administering incorrect medications and later accusing them of plotting to kill him. This heightened paranoia was accompanied by the development of a labile mood, with episodes of significant mood swings. Additionally, he displayed sexually disinhibited behavior, walking around the ward naked, and irritability at times. However, no aggression was observed at any point. Consequently, escitalopram was initially tapered to 5 mg and then discontinued, and risperidone 2 mg once daily was initiated. He received a revised provisional diagnosis of bipolar disorder, current episode manic, severe, with psychotic features.

**Figure 1:**
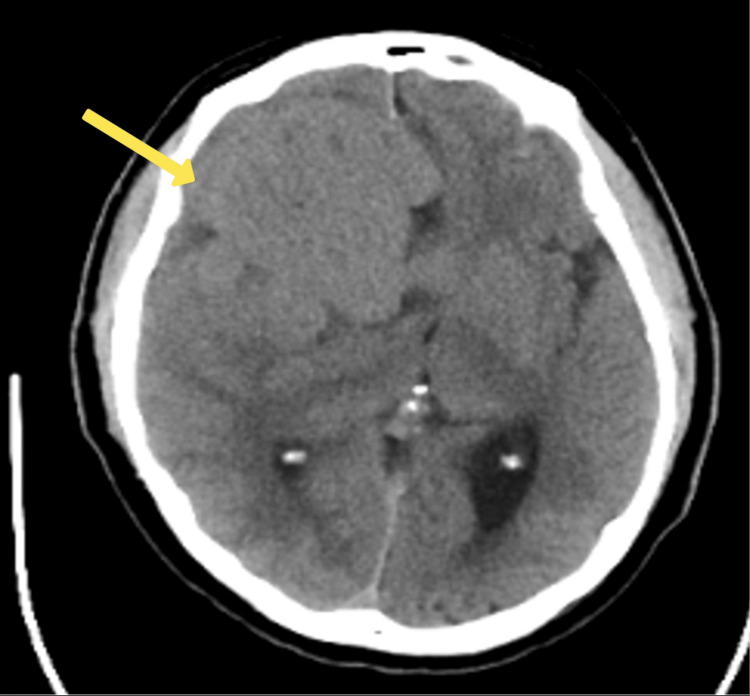
CT brain reveals a large mass (measuring approximately 6.6 x 4.7 x 6.5 cm) in the anterior cranial fossa on the right side (with associated mass effect and perilesional edema), causing erosion of the right anterior clinoid process and the roof and lateral wall of the right sphenoid sinus (see arrow). The superomedial wall of the apex of the right orbit is also eroded.

**Figure 2:**
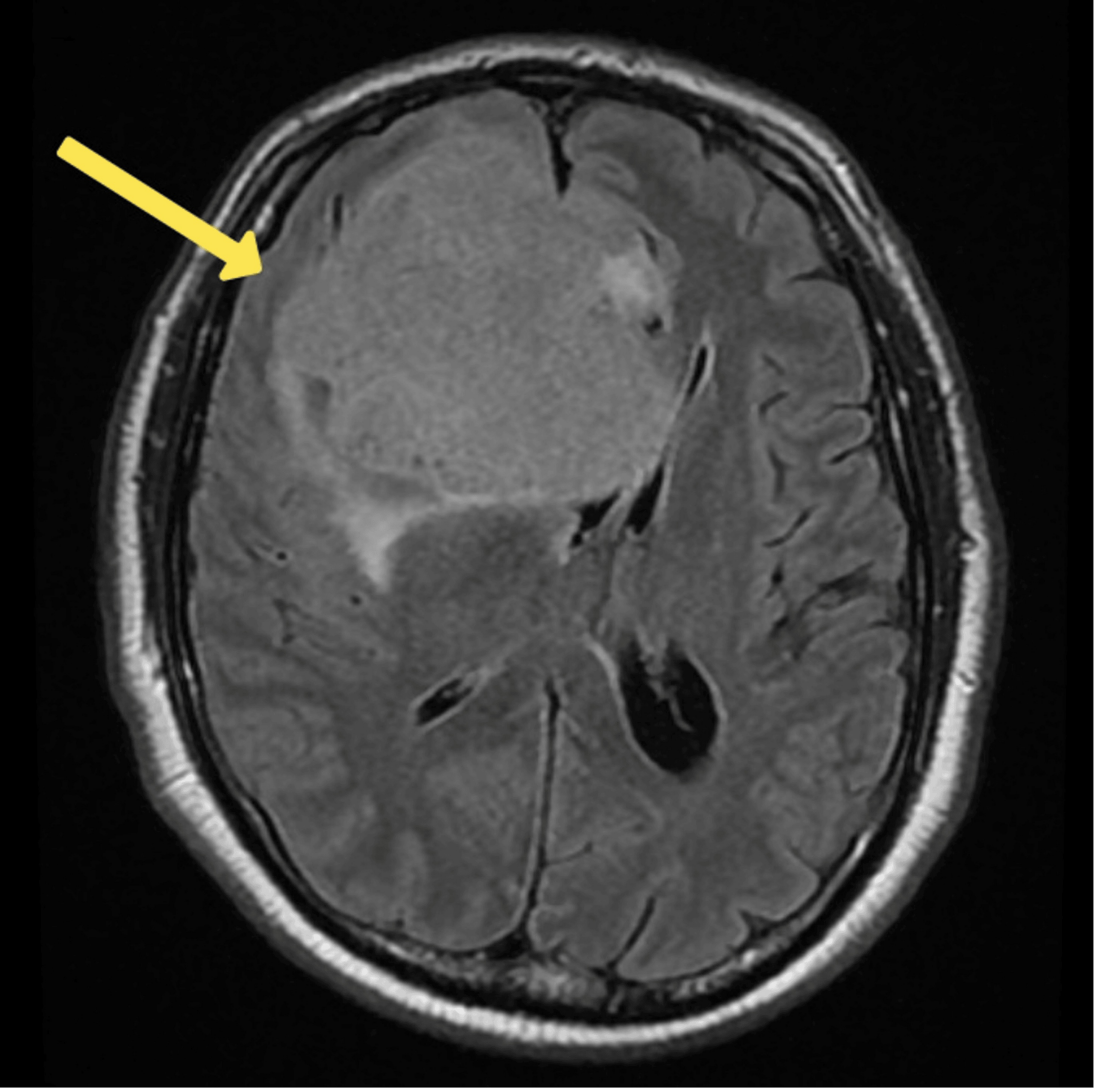
MRI brain reveals a large extra-axial mass (measuring approximately 7.1 x 7.7 x 7.5 cm) in the anterior cranial fossa on the right side (with associated mass effect and perilesional edema), causing erosion of the right anterior clinoid process, the roof and lateral wall of the right sphenoid sinus, and the superomedial wall of the apex of right orbit. The mass is seen to cause a mass effect, as evidenced by the midline shift towards the opposite side and compression of the ipsilateral lateral ventricle. The brain parenchyma is compressed and shifted superiorly. Impression: likely meningioma (see arrow).

Concerns regarding potential organic causes (due to multiple factors including, but not limited to, the patient's age being above 50 years old, this being the first onset of a psychotic episode with no past psychiatric history and no family history) led to neuroimaging studies. A CT scan (Figure 1) and an MRI (Figure 2) of the brain revealed a large extra-axial mass in the anterior cranial fossa, indicating a meningioma.

Following these findings, the patient's diagnosis was again revised to psychotic disorder due to another medical condition. He was subsequently referred to neurosurgery at his preferred private hospital for further management. His total length of stay for psychiatric treatment at our facility was 16 days.

At the private facility, he underwent a pre-op brain MRI without contrast. The findings revealed a large extra-axial tumor centered at the level of the right planum sphenoidal/optic strut and an acute small infarct involving the right side of the pons. The primary differential diagnosis included a meningioma (possibly atypical) and, less likely, other pathology, such as a solitary fibrous tumor. He thereafter underwent surgery for resection of the meningioma.

The post-op brain MRI was conducted 11 days later, following a right frontotemporoparietal craniotomy for resection of the meningioma. Subtotal resection of the right frontal region meningioma with almost complete resolution of the mass effect and midline shift was observed.

He was initiated on levetiracetam post-op as prophylaxis for potential seizures following the craniotomy. During the follow-up visit with the psychiatrist two days post-op, his MSE showed him to be alert, oriented to time, place, and person, having an appropriate appearance, normal behavior and motor activity, coherent and relevant speech of normal rate and volume, fair mood with full affect, logical thought process, with no perceptual abnormalities, and normal thought content. During another follow-up three months later, the patient remained stable, reporting a good mood, with a completely normal MSE.

## Discussion

A thorough medical history and physical examination are essential in identifying psychiatric symptoms such as personality changes, mood disturbances, anxiety, appetite changes, and unusual or treatment-resistant symptoms. Neuroimaging techniques are crucial for ruling out organic causes, such as brain tumors in patients exhibiting atypical, newly emerged, or challenging-to-treat symptoms [4]. Benign tumors such as meningiomas, which compress the frontal lobes externally, often remain asymptomatic until they grow large enough to cause noticeable changes in personality and cognitive function [6]. Frontal lobe tumors are frequently misdiagnosed or overlooked in patients with a history of alcohol abuse, depression, or personality disorders because their symptoms can mimic those of psychiatric conditions. The gradual onset of symptoms often leads clinicians to initially attribute them to depression or schizophrenia. Furthermore, once a patient receives a psychiatric diagnosis, there is typically minimal reconsideration of underlying organic causes [7].

The literature reveals various psychiatric manifestations of meningiomas, including depression, anxiety, psychosis, and personality changes. Studies have highlighted the need for early and accurate diagnosis through neuroimaging in cases of atypical psychiatric presentations. For instance, Subramoniam et al. presented a case where psychiatric symptoms were the initial indicators of meningioma, emphasizing the need for brain imaging in atypical psychiatric presentations [6]. Similarly, Zivković et al. reported a case of depression associated with convexity meningioma, which was initially resistant to antidepressant treatment but improved post-surgery [8].

Anterior skull base meningiomas are commonly linked to alterations in personality and behavior. While these tumors often impair the ventromedial prefrontal cortex (vmPFC), a region crucial for advanced cognitive functions, the specific cognitive and behavioral impacts of these meningiomas are not well comprehended [9]. Apathy, defined as a quantitative reduction of voluntary, goal-directed behaviors, is a significant symptom in patients with frontal lobe meningioma implicated in the prefrontal cortex or the basal ganglia regions. It is often misinterpreted as depression due to overlapping characteristics such as lack of motivation, decreased emotional responsiveness, and withdrawal from activities. However, apathy is more specifically characterized by diminished initiative and interest, distinct from the low mood and negative self-perception found in depression. The diagnostic challenge lies in the subtle and gradual onset of apathy, which may lead to delays in recognizing the underlying organic cause [2]. In a case report by Dautricourt et al., a patient with treatment-resistant depressive syndrome was eventually found to have meningiomatosis, highlighting the importance of considering apathy as a potential indicator of brain tumors in psychiatric evaluations [5].

Psychiatric manifestations of meningiomas are not limited to depression and apathy, as cases of psychosis, mania, and behavioral disorders have also been reported. For example, a 59-year-old man with a right parietal meningioma developed mania with psychotic features, highlighting the diverse psychiatric presentations of meningiomas [10]. Similarly, a patient with a left temporal lobe meningioma presented with generalized anxiety disorder, further demonstrating the broad spectrum of psychiatric symptoms associated with meningiomas [11]. A peculiar case of Charles Bonnet syndrome secondary to a frontal meningioma presented with visual hallucinations and depressive symptoms underscores the complexity of psychiatric manifestations in such cases [12]. Another case involved a patient with a meningioma compressing the left amygdala, presenting with anxiety and fear, illustrating how tumor location influences the type of psychiatric symptoms [13].

While meningiomas are a well-documented cause of psychiatric symptoms, other neurological conditions must also be considered in differential diagnoses. Glioblastoma multiforme, a highly aggressive brain tumor, and astrocytomas, another type of glioma, can both cause significant neuropsychiatric symptoms, including psychosis, depression, and anxiety, depending on their brain location and the extent of tissue infiltration [3,7]. Temporal lobe epilepsy (TLE), although not a tumor, can lead to psychosis, particularly when involving mesial temporal structures [4]. Pituitary adenomas, despite being benign, can cause psychiatric symptoms through pressure effects or hormonal imbalances [2]. Brain metastases from primary cancers, such as lung or breast cancer, can also present with new-onset psychosis, depending on the affected region [3]. Other potential diagnoses include cerebral abscesses, which mimic space-occupying lesions, causing confusion and psychosis [7], and neurosarcoidosis, leading to brain granulomas and psychiatric symptoms [13]. Multiple sclerosis (MS) may cause psychosis during active demyelination [11]. Limbic encephalitis, an autoimmune condition, results in severe neuropsychiatric symptoms, including psychosis [7]. Finally, normal pressure hydrocephalus (NPH), characterized by cerebrospinal fluid buildup, can manifest with cognitive decline and psychosis [9].

In patients over the age of 50 who present with new psychiatric symptoms such as psychosis, apathy, or depression, especially in the absence of a prior mental illness, clinicians should promptly consider neuroimaging to rule out organic causes such as brain tumors [1,2,5,7,8]. Early diagnosis and treatment of meningiomas can significantly improve psychiatric symptoms and overall quality of life [7,8].

One limitation of this case report is its focus on a single patient, which may limit the generalizability of the findings. Additionally, the lack of long-term follow-up data constrains our understanding of the sustained impact of treatment on psychiatric symptoms.

This case of a frontal lobe meningioma presenting with psychiatric symptoms in a late-middle-aged patient is particularly significant, as such presentations are rarely documented in the literature within Middle Eastern, Gulf, or Arab countries, highlighting the need for greater awareness of similar cases in these regions.

## Conclusions

Meningiomas often exhibit diverse psychiatric symptoms, making their early detection difficult. Clinicians should be vigilant for organic causes in patients with unusual psychiatric presentations, particularly when they do not respond to conventional treatments. Neuroimaging is essential for the prompt diagnosis and effective management of these cases, which can improve patient outcomes. Meningiomas, the second most common central nervous system tumors, grow slowly and can be asymptomatic for years, often discovered incidentally. Due to their frequent psychiatric manifestations, early diagnosis is challenging. For patients over 50 years old with atypical psychiatric symptoms, neuroimaging should be considered to exclude organic causes. Timely diagnosis and intervention are crucial for better patient outcomes, and the management of incidentalomas such as meningiomas will be increasingly important in clinical practice.
